# Membrane Processes for Microplastic Removal

**DOI:** 10.3390/molecules24224148

**Published:** 2019-11-15

**Authors:** Teresa Poerio, Emma Piacentini, Rosalinda Mazzei

**Affiliations:** National Research Council—Institute on Membrane Technology (ITM–CNR), c/o University of Calabria, Cubo 17C, Via Pietro BUCCI, 87036 Rende (CS), Italy; e.piacentini@itm.cnr.it (E.P.); r.mazzei@itm.cnr.it (R.M.)

**Keywords:** plastic removal, wastewaters treatment, membrane processes, ultrafiltration, dynamic membranes, reverse osmosis, membrane bioreactors, membranes reuse, membranes recycling

## Abstract

Plastic pollution of the aquatic environment is a major concern considering the disastrous impact on the environment and on human beings. The significant and continuous increase in the production of plastics causes an enormous amount of plastic waste on the land entering the aquatic environment. Furthermore, wastewater treatment plants (WWTPs) are reported as the main source of microplastic and nanoplastic in the effluents, since they are not properly designed for this purpose. The application of advanced wastewater treatment technologies is mandatory to avoid effluent contamination by plastics. A concrete solution can be represented by membrane technologies as tertiary treatment of effluents in integrated systems for wastewater treatment, in particular, for the plastic particles with a smaller size (< 100 nm). In this review, a survey of the membrane processes applied in the plastic removal is analyzed and critically discussed. From the literature analysis, it was found that the removal of microplastic by membrane technology is still insufficient, and without the use of specially designed approaches, with the exception of membrane bioreactors (MBRs).

## 1. Introduction

The world plastic production is constantly growing, with production rising from 335 million tons in 2016 to 348 million tons in 2017. [1]. Asia is the largest producer of plastics (50.1%), followed by Europe (18.5%), North American Free Trade Agreement (17.7%), Middle East, Africa (7.71%), Latin America (4%) and Commonwealth of Independent States (2.6%). This significant increase and widespread in worldwide production of plastics produces a huge amount of plastics waste on land that enters the aquatic environment causing growing concerns [2]. 

Different papers report that the presence of a large part of microplastic fibers in the aquatic environment is due to the washing of synthetic clothes [3,4]. The ingestion of microplastic, besides causing the obstruction of digestive tract, can facilitate the transfer of contaminants adsorbed by the plastic, with unclear consequences to the health of aquatic organisms and humans [5,6,7]. Indeed a major problem with microplastic is their ability to adsorb other common environmental contaminants, such as metals [8,9,10], pharmaceuticals [11,12], personal care products [12] and others [13,14]. Consequently, the microplastic can potentially cause diseases such as cancer, a malformation in animals and humans, impaired reproductive activity, and reduced immune response [15].

Microplastic removal from the aquatic environment represents a new urgent challenge in the last decade considering the disastrous impact for aquatic species and human beings. These contaminants have been detected in various aquatic environments such as lakes, rivers, oceans, urban wastewater effluents. 

Based on the particle size, plastics are defined as microplastic (MP) and nanoplastic (NP). 

According to the National Oceanic and Atmospheric Administration (NOAA), the definition of microplastic is particles of synthetic polymers (less than 5 mm in diameter), which resist (bio) degradation [16]. On the other hand, nanoplastic is defined as particles (nanospheres, nanowires/nanotubes, and nanofilms) with smaller dimensions, between 1 and 100 nm [17,18,19,20].

Based on their origin, MP and NP are divided into two classes, primary and secondary plastic [21]. The first includes small pieces of specially manufactured plastic, such as hand and facial cleansers, shower gels, toothpaste, industrial scrubbers, and plastic micro-nanospheres, etc., while the latter are small pieces of plastic derived from the deterioration of larger plastic waste both at sea and on land. 

The more common plastic materials founded in the effluents are polypropylene (PP), polyethylene (PE), Polystyrene (PS), polyvinyl-chloride (PVC), polycarbonate (PC), polyamides (PA) Polyester (PES), and polyethylene terephthalate (PET), these are reversible thermoplastic polymers, highly recyclable materials that can be heated, cooled, and shaped repeatedly [22]. 

In addition to these recyclable materials, also thermoset polymers have been found, they are irreversible materials that after being heated and formed they cannot be re-melted, reformed, and recycled; the most common thermoset materials are epoxy resins, vinyl ester, silicone, melamine resin, unsaturated polyester, phenolic resins, polyurethane, formaldehyde, acrylic resins, etc. [22]. 

**Figure 1:**
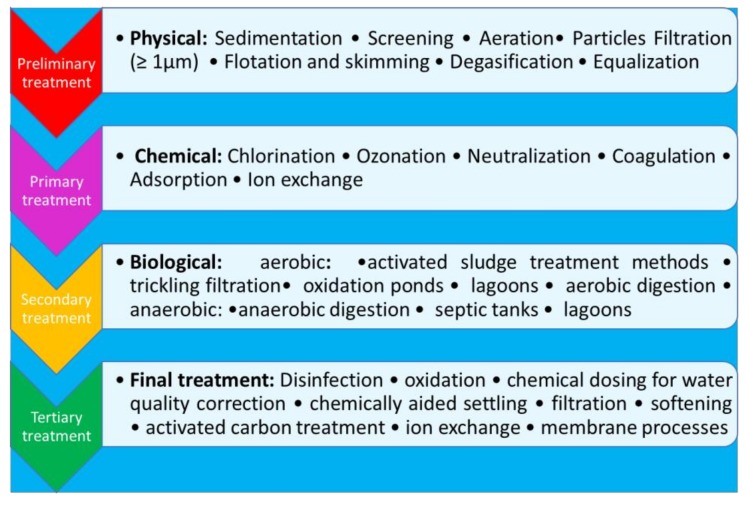
Classification of wastewater treatment methods.

Nowadays, 98% of MP is retained from wastewater treatment plants (WWTPs) but MP with a size smaller than 20 μm and NP is not retained; therefore, WWTPs plants are supposed to be one of the major responsibility for the plastic pollution in wastewater effluents [22,23,24]. The wastewater processing can be grouped into four main treatments: preliminary treatment, primary treatment, secondary treatment, and tertiary treatment, also named final or advanced treatment [25] (Figure 1). 

These treatments include different methods and technologies (some of them are reported in Figure 1) whose use depends on the nature of the wastewater to be treated, as well as on the different quality of the water to be obtained (drinking water, swimming, agriculture, industries, etc.). 

Preliminary treatment is often required to protect equipment and enhance the performance of subsequent treatment processes. Primary treatment consists of removing large suspended organic solids, but the liquid effluent coming from this treatment still contains a large amount of suspended organic material, indeed the microplastic removal efficiency is approximately 25%. The secondary treatment, despite more efficient, can reduce microplastic concentrations by 75% [26]. Removal efficiency of 98% could be reached by the not always used tertiary treatment, producing an effluent of almost drinking-water quality [23,24,25,26]. The limited application of tertiary treatments in the WWTPs, coupled with the huge amount of treated wastewaters to obtain water with different quality, are a source of plastic in the effluents. The application of advanced final stage wastewater treatment technologies is mandatory to avoid effluent contamination by plastics. Among the tertiary treatment processes, membrane operations can offer an effective solution to the microplastic and nanoplastic pollution in the effluents. 

To date, only a few papers report the application of membrane processes for microplastic removal. In this review, an analysis of the documents in this field has been carried out to highlight the growing interest of the scientific community towards the problems of plastic pollution as well as to demonstrate the still insufficient knowledge and experience in the removal of plastic, with specific emphasis on the application of the membrane technologies. The membrane processes actually applied to plastic removal have been reported and critically discussed.

## 2. Literature Analysis on the Microplastic and Its Removal by Membrane Processes 

**Figure 2:**
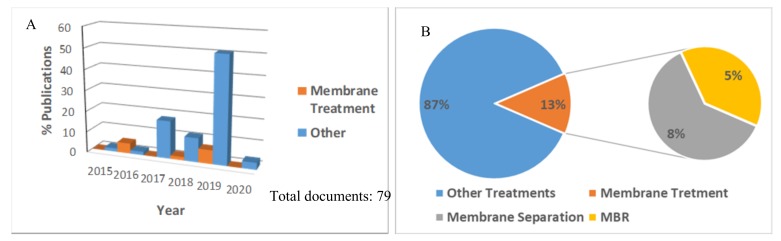
(**A**) The distribution of publications related to microplastic contaminant removal from 2015 to 2020 and (**B**) the incidence of the research on membrane technology applications with respect to other existing treatment processes.

A document search was conducted using Scopus (https://www.scopus.com) searching “microplastic removal” as a specific keyword in “Article Title, Abstract, Keywords” and selecting “Article” as document type in order to perform the search as general as possible, excluding the review. A total of 79 documents published from 2015 to 2020 were collected and used as the database for analysis. The chronological distribution of publications related to the microplastic removal by membrane technology and other treatments, as well as the incidence of membrane treatments in the field, are reported in Figure 2.

In the last five years, the removal of microplastic received increased attention, and the distribution in Figure 1 shows one peak corresponding to 2019. Although the application of membrane technology in the removal of microplastics is still limited, the last year registers a certain increase of studies in which the conventional membrane separation process and membrane bioreactor (MBR )are combined with the other existing treatment processes to reach a more effective removal of microplastic contaminants from wastewaters. The removal of the plastic was found to strictly depend on some parameters used as indicators to classify it, such as the shape, size, and mass of plastic particles. The main influencing factors that can affect the performance of membrane processes for MP removal are listed in Table 1.

It should be highlighted that the comparison of the literature data in terms of removal efficiency of MP is not so simple considering the different composition of the treated wastewater, the different characterization procedures of MP, and also the range size of the MP considered. In particular, the inexistence of standardized protocols of characterization has led to a lot of information not directly comparable due to the use of different units (e.g., mass per volume, number per volume, etc.) [19]. The removal efficiency of microparticles in some WWTPs was reported by Sun et al. 2019 [27] and summarized in Table 2. In some cases, a very high MP removal was reported, probably due to the size range of the MP considered. However, despite the high plastic removal, the huge amount of treated wastewaters is considered a source of plastic in the effluents [22].

**Figure 3:**
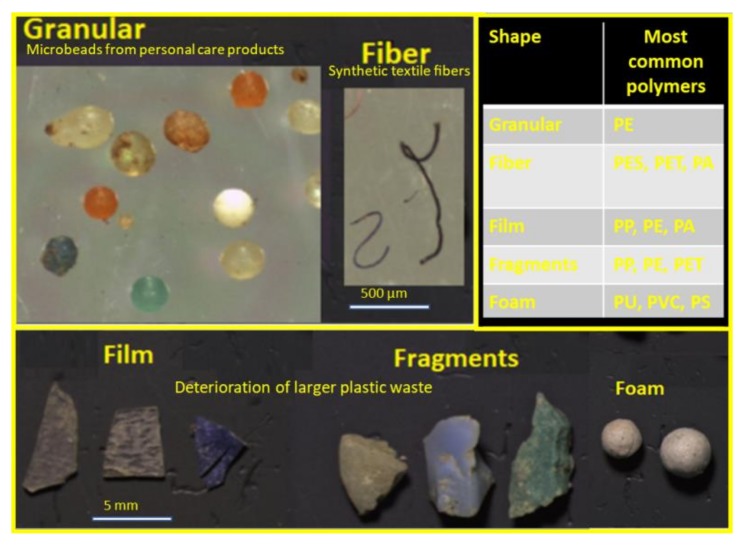
Common microplastic shapes and related materials (Elaborated from [19,22]).

The shape of the plastic particles affects their removal efficiency in WWTPs and can determine the interaction between other contaminants or microorganisms [28]. The shape is categorized as fiber, granular, fragment, film, and foam (Figure 3). 

The most abundant shape of plastic particles found in the wastewaters is into fiber due to the discharge of domestic washing machines containing synthetic polymers for clothing [29]. Some studies report that pretreatment removes fibers more effectively than fragments, while secondary treatment removes more fragment particles than fibers [27,28,29,30,31].

Regarding the plastic size dimensions, 25 µm, 100 µm, and 500 µm are most frequently used for size classification [21,30,31], but recent studies showed that plastic particles with nanometer size are also present in the wastewater due to the fragmentation of synthetic fibers or to the degradation of polymers [19,32]. Fragmentation and degradation are the two main steps in the breakdown process of polymer particles [33]. Fragmentation is the process of breakdown of larger polymer chains into smaller polymer fragments. Degradation is a process of bond-break, followed by a chemical transformation that changes the polymer properties. This process can occur by hydrolysis, photodegradation, mechanical/physical degradation, thermooxidative degradation, and biodegradation [34]. The reduced size allows the nanoparticles to pass through biological barriers [35] to penetrate tissue, to affect the behavior and metabolism of organisms to accumulate in organs, and to enter the base of a food chain more easily than larger. Furthermore, the degradation processes increase the surface area of its degradation products, causing an enormous biological impact, for example, the 40 nm nanoparticles deriving from the degradation of a common plastic bag have a surface area of 2600 m^2^ [36]. A more detailed physical-chemical characterization of plastic, such as size, mass, shape, and chemical composition is necessary, since not only can it help for a better understanding of their real threat [19,20] but it can also allow selecting appropriate methodologies that guarantee a more efficient plastic removal from effluents.

Regarding the amount of microplastic contained in wastewater, some information was found in studies conducted in Northern Europe, the United States, and Australia. The detected amounts of microplastic varied between 1–3160 and 0.0007–125 particles L^−1^ for raw and treated wastewater, respectively. However, data for the Mediterranean area, Asia and South America are lacking [37]. Jenna R. Jambeck et al. calculated that 275 million metric tons (MT) of plastic waste were produced in 192 coastal countries in 2010, with 4.8 to 12.7 million MT entering the ocean. Furthermore, this quantity of plastic waste is expected to increase by order of magnitude by 2025 [38].

### 2.1. Ultrafiltration

Ultrafiltration (UF) represents a feasible alternative for the water treatment since it permits to attain drinking water of high quality in an economic manner thanks to low energy consumption, high separation efficiency, and compact plant size [39,40,41]. It is a low-pressure process (1–10 bar) that, using asymmetric UF membranes having a pore size between 1–100 nm, can reject particulates and macromolecules such as proteins, fatty acids, bacteria, protozoa, viruses, and suspended solids. In particular, it can reduce organic matter and BOD (biological oxygen demand) by at least 95%, greatly reduce turbidity, exceed regulatory standards of water quality, achieving 90–100% pathogen removal. Moreover, many municipal water treatment facilities use UF treatment against contamination from cryptosporidium, giardia, and other organisms that could cause serious illness if ingested [42,43]. Therefore, UF is used to replace existing secondary (sedimentation, flocculation, coagulation) and tertiary filtration (sand filtration and chlorination) methods employed in WWT. In particular, it can permit the reuse of water coming from the industries that consume huge volumes of water or discharge highly toxic effluent such as chemicals, steel, plastics and resins, paper and pulp, pharmaceutical and the food and beverage industries, water and wastewater treatment plants, and etc. [39].

UF, despite a broad molecular weight cut off (MWCO) range, is less active in removing low molecular weight organic matters. In many cases, UF is integrated into the process, using primary (flotation and filtration) and some secondary treatments as pretreatment stages and used for pre-filtration in reverse-osmosis plants to protect the reverse-osmosis process. 

Today, the ultrafiltration process coupled with the coagulation step is one of the main water treatment technologies in the current water plants proving a significant removal of organic matter in water. However, these technologies are not properly designed for the microplastic removal that remains in the final effluents [22,44]. Indeed, most of the papers report the removal of the natural organic matter (NOM) that is a complex matrix of organic compounds with a wide variety of chemical properties, chemical composition, and molecular weight [45,46]. 

However, the worrying levels of microplastic in freshwaters make a mandatory a-depth investigation of the behavior of microplastic removal during coagulation and ultrafiltration (UF) processes, also considering that they are water treatment technologies used in the production of drinking water [47,48].

**Figure 4:**
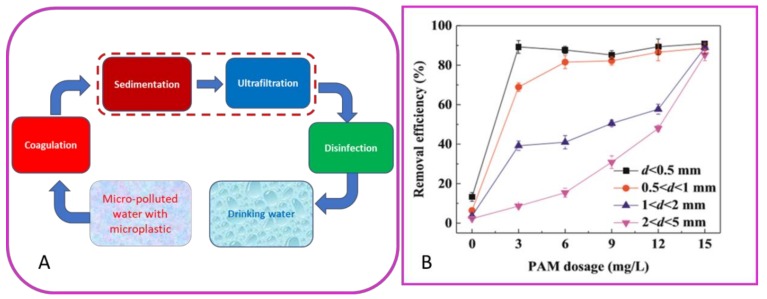
Scheme of the process for removal (**A**) and removal efficiency (**B**) of polyethylene (PE) using FeCl_3_·6H_2_O and anionic polyacrylamide (PAM)(elaborated from Ma et al. 2019).

To date, a few papers report the study of microplastic removal by coagulation and UF process for the production of drinking water [49,50]. In particular, the study reported by Ma et al. 2019 focused on the removal behavior of polyethylene (PE) in drinking water treatment by ultrafiltration and coagulation processes by using an Fe-based coagulant. PE is the most abundant plastic pollutant detected in the water, and moreover, its density (0.92–0.97 g/cm^3^) very close to that of water makes it difficult to remove by water treatment processes. Low removal efficiency of PE particles (below 15%) was observed after coagulation, indicating the ineffectiveness of the sole coagulation process with respect to microplastic removal. However, when the Polyacrylamide (PAM) was added to enhance the coagulation performance, removal efficiency of small-particle-size PE (d < 0.5 mm) significantly increased from 13 to 91% (Figure 4). 

For what concerns the UF performance, an interesting result was that the membrane fouling was progressively reduced after coagulation with PE. In particular, by increasing the dosage of coagulant, the porosity of the floc cake layer increased due to the presence of PE particles, especially the large ones. As a result, less severe membrane fouling was induced compared to that with flocs alone. The presence of larger PE particles had positive effects on the membrane fouling. The membrane flux decreased by only 10% in the presence of large-particle-size PE (2 < d < 5 mm) after the coagulation with 0.2 mmol/L PAM and 2 mmol/L FeCl_3_·6H_2_O, respectively [50]. However, this behavior may not be a general rule but can depend on different parameters related to the membrane process, as well as to the plastic characteristic (chemical composition, size, and shape).

The final comment is that as a general principle, UF could be used to remove PE particles totally, but many efforts are required to understand how the cake layer formation and then the fouling is influenced by the presence of plastic particles. Indeed, also the shape of microplastic can affect their removal in different water treatments;. As reported in Talvitie et al. 2017, a portion of plastic in “fiber shape” is not retained by the WWTPs. Therefore, the final stage treatments have to be properly designed for the removal of the fiber to increase the removal efficiency of plastic. 

### 2.2. Dynamic Membranes Technology

Recently, DM is emerging as an attractive technology for municipal wastewater treatment [51,52], surface water treatment [53], oily water treatment [54], industrial wastewater treatment [55], and sludge treatment [56]. 

This technology is based on the formation of a cake layer (dynamic membrane, DM), which acts as a secondary membrane/barrier created when particles and other foulants in the wastewater are filtered through a supporting membrane. Since the filtration mechanism of the DM is quite different compared to the MF/UF processes, in the sense that the fouling and foulants are necessary to create the DM layer, the resistance to filtration is caused exclusively by the layer of the cake. However, thicker layers and dense fouling cause a loss of membrane performance. The parameters that must be taken into consideration to limit the formation of fouling are the same that are involved in the DM formation [57].

The DM formation process depends on various parameters relating to the supporting membranes (membrane materials, membrane pore size), to the deposited material (particle size, concentration) and to the operating conditions (operating pressure, cross-flow velocity) [52]. 

DM technology attracted great attention because:

(i) it employs relatively lower-cost materials compared to traditional membranes (such as mesh, non-woven fabric, and woven filter cloth, and stainless-steel mesh);
(ii) extra chemicals or other contaminants are not introduced considering that the filtration layer is formed by the contaminants of the influent;
(iii) the experimental setup is generally more compact than the traditional membrane processes (e.g., for ultrafiltration (UF) and microfiltration (MF)), since DM permeation flux is much larger, the membrane module quantities are saved;
(iv) the energy supply is lower since DM operates under gravity driving mode, and lower transmembrane pressure is required compared to traditional membranes.

The application of DM technology for micro-plastics removal has been also studied [58] because DM is suitable to remove low-density/poorly settling particles. DM technology was applied for micro-particle removal from synthetic wastewater under a gravity-driven operation by using a lab-scale DM filtration setup. The DM was formed on a 90 μm mesh and the synthetic wastewater was prepared with diatomite (AR, Tianjin BASF, D90 = 90.5 μm, meaning that over 90% of the particles in this study are within the defined size range of micro-particles) and tap water. The effluent turbidity was reduced to <1 NTU (Nephelometric Turbidity Unit) after 20 min of filtration, verifying the effective removal of micro-particles by the DM. The transmembrane pressure (TMP) during the DM filtration process (in the range from 80 to 180 mm of water) was lower than that observed for conventional microfiltration and ultrafiltration (16 times lesser than the value obtained for wastewater microfiltration) also reducing the energy consumption. Different influent flux was used (in the range from 9 to 21 L/h), and the linear increase of TMP (at a constant rate) was observed with DM filtration time. At an influent flux of 9 L/h, the effluent turbidity was 4.94 NTU at 10 min and 1.41 NTU at 20 min of filtration time while, at an influent flux of 21 L/h, the effluent turbidity was reduced to 7.14 NTU after 3 min and 1.53 NTU at 5 min of filtration indicating that a higher influent flux facilitated the rapid formation of the DM. The DM formation process was strongly affected by the influent particle concentration. Higher influent particle concentrations resulted in more microparticles being filtered through the supporting mesh, laying the foundation for the rapid formation of the DM layer and a faster effluent turbidity reduction. As a result, increasing flux and influent particle concentration can be used for the control of the DM formation process.

### 2.3. Reverse Osmosis

Reverse Osmosis (RO), is actually used in municipal and industrial water treatment systems to purify water using nonporous or nanofiltration membranes (pore size > 2 nm) by removing salts, contaminants, heavy metals, and other impurities. It works by applying a high pressure (10–100 bar) to a concentrated water solution that forces the water through a semipermeable membrane, leaving all the other substances essentially in a more concentrated water solution. It is currently applied also in food and beverage production, biopharmaceutical manufacturing, power generation, production of high purity water, and desalination of brackish waters and seawater, as well in the recovery of industrial and municipal wastewater [59].

RO membrane fouling is a major challenge for reliable membrane performance [60]. A pretreatment stage is mandatory to maintain the flux rates, to control membrane fouling at industrial scale RO desalination systems, minimizing the membranes cleaning frequency, and prolong the useful life of the RO equipment. Usually, some common pretreatment involves the use of chemicals such as coagulants, antiscalants, oxidizing agents, and disinfectants [60,61]. Other strategies for fouling mitigation include cleaning, surface modification, and the use of novel membrane materials [61]. Nowadays, steady performance in terms of water quality and flux was achieved by the combination of an UF pretreatment with RO in the desalination process [62]. A growing trend in the application of combined RO-UF plants for desalination at an industrial scale is reported by Ashfaq et al. 2019. Some plants are reported here: Tuas, Singapore (Capacity: 318,000 m3/day; Year: 2013), Ashdod, Israel (Capacity: 275,000 m^3^/day; Year: 2013), Ajman, United Arab Emirates (Capacity: 115,000 m^3^/day; Year: 2012), Tangshan, China (Capacity: 110,000 m^3^/day; Year: 2012), Teshi, Ghana (Capacity: 60,000 m^3^/day; Year: 2014), Accra, Ghana (Capacity: 60,000 m^3^/day; Year: 2014), Red Sea Coast—Saudia Arabia (Capacity: 30,000 m^3^/day, Year: 2016), Gwangyang, South Korea (Capacity: 30,000 m^3^/day; Year: 2015) [63].

The performance of the RO process with respect to MPs removal was reported by Ziajahromi et al. 2017 [31]. They characterized and quantified the microplastic in samples coming from a WWTP that produce a highly treated effluent, including screening and sedimentation, biological treatment, flocculation, disinfection/de-chlorination processes, ultrafiltration, and finally a reverse osmosis (RO) process. Results indicate the presence of microplastic fibers in the samples after RO process. In particular, irregular shaped microplastic were detected and identified by Fourier transform infrared spectroscopy analyses in attenuated total reflectance (ATR-FTIR) as alkyd resin (modified polyester) commonly used in paints. This microplastic detection was attributed by the authors to the occurrence of some membrane defects or simply small openings between pipework, indicating the necessity to ad-hoc design the processes for microplastic removal.

Most of the more performant applications of RO in the microplastic removal are obtained when coupled with membrane bioreactor technology that is hereafter discussed.

### 2.4. Membrane Bioreactor (MBR)

Membrane bioreactor (MBR) are systems in which catalysis promoted by biological catalysts (bacteria, enzymes), is coupled to a separation process, operated by a membrane system (generally microfiltration or ultrafiltration) [64].

Thanks to the different compartments created by the membrane, a controlled heterogeneous (organic/water)/multiphase (liquid/gas) reaction system can be developed. The different phases can be kept separated (as for example, in a membrane-based solvent extraction process), or they can be dispersed into each other (as in a membrane emulsification process). Besides, the versatility of the technology permits an easy integration with other process (e.g., pervaporation, reverse osmosis) perfectly in line with green chemistry principles, within the logic of process intensification, which offers new and much more opportunities in terms of competitiveness, product quality improvement, process or product novelty and environmental friendliness [65].

Nowadays, MBR is deemed as one of the most powerful technologies for efficient municipal and industrial wastewater treatment around the world; however, new emerging fields of application are vastly growing, such as food, pharmaceutical, biorefinery, and biodiesel production [66].

In these last years, MBRs received an extensive academic interest and a very rapid growth in practical municipal and industrial wastewater treatment applications. The great success is given by the significant improvement given by this technology, respect to the traditional methods of water treatment, such as high effluent quality, small footprint, complete separation of hydraulic retention time (HRT), and solids retention time (STR), easy scale-up, etc. Regarding fouling control, various methods have been developed in this technology. The most recent methods are based on mechanically assisted aeration scouring, in-situ chemical cleaning enzymatic and bacterial degradation of foulants electrically assisted fouling mitigation, and nanomaterial-based membranes [67]. The demonstration of the exponential interest of this technology is the significant increase on both large (≥10,000 m^3^/d) and super large-scale (≥100,000 m^3^/d) plants worldwide. The Beijing Wenyu River plant in China was the first to be built, with the capacity of a super-large scale (100,000 m^3^/d) and subsequently many new plants were built in China (around 200) and in all the world and at the following link is possible to see them https://www.thembrsite.com/largest-membrane-bioreactor-plants-worldwide. It is expected that the plant under construction in Sweden (Henriksdal plant) will be the largest MBR in the world with a treatment capacity of 864,000 m^3^/d [64,65].

**Figure 5:**
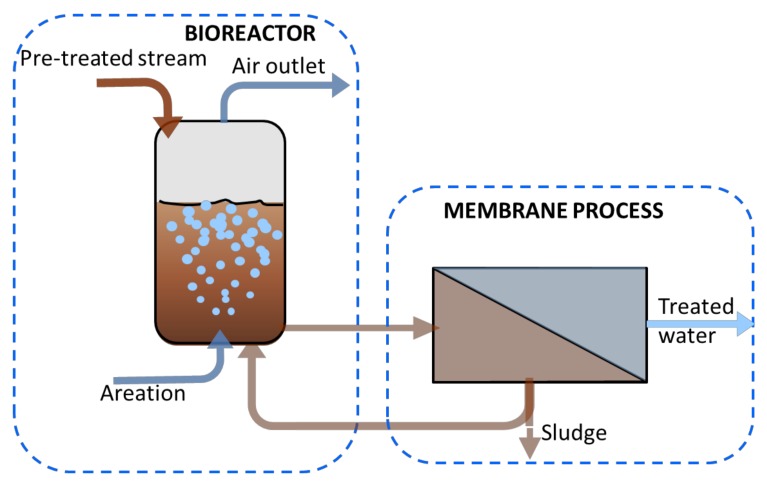
Schematic representation of a MBR process.

In MP treatment, the role of MBR is the decrease of solution complexity by the biodegradation of the organic matter; this will permit the purification of MP and its further treatment. The process generally starts when a pre-treated streams enters in the bioreactor, where the process of biodegradation of organic matter is carried out. The produced mixed liquor is then pumped along with semi-crossflow filtration system for the separation process (Figure 5). Thanks to the membrane process, the MP is concentrated in the retentate stream.

In recent work [68], the performance of MBR was compared with other final-stage wastewater treatment technologies (disc-filter, rapid sand filtration, and dissolved air flotation) for MP removal. The MBR used is located in Finland and consisted of a submerged membrane unit (8 m^2^, 0.4 µm) and ultrafiltration process.

**Figure 6:**
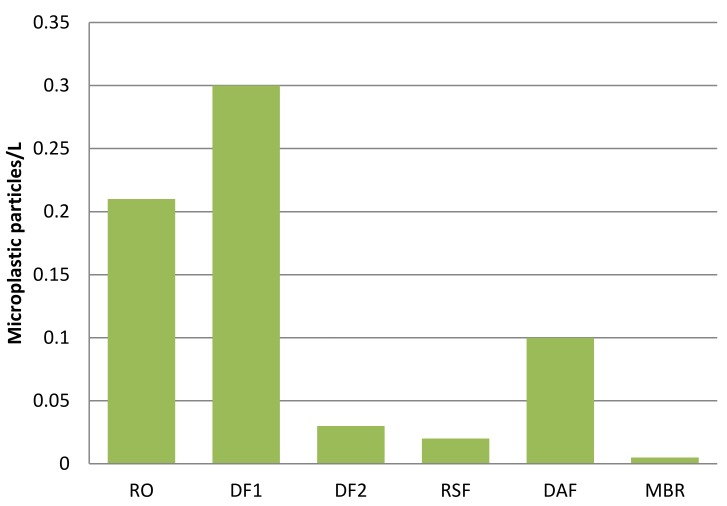
The number of microplastic particles per liter in the final effluent of each wastewater treatment plant (Data elaborated from Ziajahromi et al. 2017 and Talvitie et al. 2017). RO: Reverse Osmosis; DF1: Disc Filter with pore size 10 µm; DF2: Disc Filter with pore size 20 µm; RSF: Rapid Sand Filters; DAF: Dissolved Air Flotation; MBR: Membrane Bioreactor.

Respect to the other advanced treatment processes, the used MBR showed a significant improvement in MP removal (99%), higher quality of final effluent, and a great potentiality in decreasing the number of process stages, replacing the conventional secondary clarifiers (conventional activated sludge, CAS). A comparison among the tertiary treatments is shown in Figure 6. From this figure, it is possible to assess that MBR allowed the highest reduction of MP in the final effluent, demonstrating that the membrane-based technology is the most efficient.

However, although MP in the wastewater treatment could be removed through different wastewater treatment plants (which in some cases include a step of MBR) [22], any full-scale plant has been developed, or specifically designed for this purpose. So, tuned MP treatment technologies or strategies which can support MP removal in existing wastewater treatment plant are currently at the preliminary stage of research.

Among the recently developed strategies, the use of a mixture of different enzymes, as a preliminary “plastic preserving” maceration step, seems to be very promising. The aim of “plastic preserving enzyme maceration” is the degradation of all the organic matter in order to produce a purified MP solution for further treatment [27].

The biodegradation is an alternative strategy in which MBR was used for plasticizer treatment. Different studies have indicated the possibility of complete phthalate esters degradation by a wide class of bacteria and action mycetes [69,70,71]. The most well-known bacteria for phthalate esters degradation are reviewed in Gao et al. (2016) [70].

Also, in this case, the combination of biodegradation in the integrated process seems more appropriate than individual treatment step. At present, MBR technology in phthalate esters biodegradation is studied in the laboratory alone or coupled with activated sludge, starting from different wastewater origin (synthetic [72], paper mill wastewater [73], municipal solid waste leachate [74], etc.). In WWTP, the use of MBR showed about 70% higher removal of Di(2-ethylhexyl) phthalate (DEHP) of conventional treatment (3%) [72] further improved (83%) if associated with preliminary adsorption. A complete MP degradation was reached if MBR was associated with a preliminary anaerobic treatment and followed by an RO filtration [73].

However, the biodegradation efficiency in the MBR is also strictly related to physicochemical properties of the phthalate esters and operative conditions such as hydraulic retention time (HRT), initial feed concentration, etc.

A very promising discovery, which could be in the future associated with MBR technology, is the isolation of a novel bacterium (Idonella sakaiensis) able to use polyethylene terephthalate (PET) as its major energy and carbon source [75]. In particular, this bacterium produces two different enzymes, when in contact with PET, which can efficiently convert PET in the less dangerous monomers (terephthalic acid and ethylene glycol). Dawson and co-workers (2018), recently reported in Nature Communications the size reduction of microplastic (from 31.5 µm to less than 1 µm) when exposed to Antartic Krill (Euphasia superba) [76]. An in-depth study of the mechanism by which Antartic Krill can reduce the size of MP will show the enzyme complex capable of performing this process. The enzymes can be easily integrated with the MBR, so in the future, it will probably be possible to degrade the MP in the enzymatic membrane reactor, as already demonstrated by Barth et al. for PET degradation [77].

### 2.5. Polymeric Membranes as Source of Plastic Waste: Recent Advances in Their Reuse and Recycling

Nowadays, membrane technology is widely used in water and wastewater treatment with a well-established market. The world market for Membrane Filtration is expected to grow over the next five years, reaching 7030 million US$ in 2024, from 4710 million US$ in 2019, according to a new GIR (Global Info Research) study [78]. The huge spread of membrane processes has raised the need to develop methods to reuse and recycle these materials [79]. Many efforts have been made the LIFE+ TRANSFOMEM research project (LIFE13 ENV/ES/000751) [80], “Transformation of disposed reverse osmosis membranes into recycled ultra- and nanofiltration membranes”, in which the recycling process of disposed reverse osmosis membranes and their reuse in nanofiltration and ultrafiltration processes have been studied. Project results demonstrated that almost 70% of the membranes are recyclable, and the use of recycled membranes can save between 85% and 95% compared to the acquisition of new commercial membranes. Furthermore, there is a company called “MemRe, RO Membrane Recycling”, based in Germany, which deals with the recycling and reuse of membranes at the end of their life [81]. In addition, it should also be noted that the production of membranes is increasingly oriented to the use of new bio-based polymers (recyclable and biodegradable) as an alternative to petrochemical polymers [82].

## 3. Conclusions

The analysis of the literature has shown that ad hoc designed microplastic treatment processes must be developed to limit plastic pollution. The water industry and the WWTPs do not currently have experience or technologies to efficiently separate MP from effluents. From several papers, it emerged that advanced tertiary treatment is needed to remove the plastic for wastewater. Among the tertiary processes, the membrane processes, MBR in particular, appear to be the most promising with MP removal of 99.9%, also offering the possibility to decrease the number of process stages in the WWTPs.

Furthermore, a more detailed and uniform chemical-physical characterization of the plastic is mandatory to select appropriate methodologies that guarantee a more efficient removal of the plastic from the effluents. From literature emerged that the data are not easily comparable to each other due to the lack of standardized characterization protocols. This characterization should also include nanoplastics that could have a more serious biological impact.

The constructive actions for the reduction of pollution from microplastic that can act in synergy are the implementation of an environmental pollution awareness policy that leads to a reduction in the use of single-use plastic materials and the design of operational processes and production based on the use of biodegradable materials to prevent accumulation in the environment.

## Figures and Tables

**Table 1:** Influencing factors and membrane process parameters to be considered for microplastic (MP )removal by membrane processes.

	Influencing Factors	Membrane Process Parameters
**Membrane process**	Membrane material	-Flux -Transmembrane pressure (TMP) -Polarization concentration -Cake layer formation and fouling -Rejection/Removal -Specific energy consumption (SEC)
	Membrane pore size	
	Membrane thickness	
	Membrane surface properties	
	Source of polluted water (Seawater, surface water, municipal water, industrial wastewater etc.)	
**Micro-Nanoplastic**	Shape	
	Size	
	Mass	
	Chemical composition	
	Concentration	

**Table 2:** Microplastic removal by different wastewater treatment plants (WWTPs) (Data elaborated from Sun et al. 2019).

Treatment Processes	Microplastic Removal (%)	WWTP location
Primary, Secondary	99.9	Sweden
Primary, Secondary (Biofilter)	88.1	France
Primary, Secondary	99.9	United States
Primary, Secondary	98.4	Scotland
Primary, Secondary	11–94	Netherlands
Primary, Secondary	95.6	United States
Primary, Secondary	98.3	Finland
Primary/AnMBR	99.4	United States
Primary/MBR	99.3	Finland
Primary, Secondary, Tertiary (GF)	97.2	United States
Primary, Secondary, Tertiary (BAF)	97.8	Finland

Secondary treatment: conventional activated sludge process; AnMBR: anaerobic membrane bioreactor, MBR: membrane bioreactor; GF: granular filter; BAF: biological aerated filter.
